# miR-107 affects cartilage matrix degradation in the pathogenesis of knee osteoarthritis by regulating caspase-1

**DOI:** 10.1186/s13018-020-02121-7

**Published:** 2021-01-11

**Authors:** Jiatian Qian, Peiliang Fu, Shiao Li, Xiang Li, Yancheng Chen, Zhenen Lin

**Affiliations:** 1 Joint Divison of Orthopedic Department, Changzheng Hospital, Shanghai, 200003 China; 2 Department of Orthopaedics, Fuzhou Second Hospital Affiliated to Xiamen University, No. 47 Shangteng Road, Cangshan District, Fu Zhou, 350007 Fujian China

**Keywords:** miR-107, Cartilage matrix, Knee osteoarthritis, Caspase-1

## Abstract

**Background:**

Knee osteoarthritis (KOA) seriously affects the quality of life of KOA patients. This study aimed to investigate whether miR-107 could regulate KOA through pyroptosis to affect collagen protein secreted by chondrocytes through IL-1β.

**Methods:**

The proliferation of chondrocytes was detected by CCK-8 assay. RT-qPCR analysis was used to identify miR-107 expression and transfection effects. The expression of Col II, IL-1β, IL-18, and MMP13 in supernatant of chondrocytes or chondrocytes was detected by ELISA assay and western blot analysis. The pyroptosis of chondrocytes was analyzed by TUNEL assay and the expression of pyroptosis-related proteins was analyzed by western blot. Luciferase reporter assay confirmed the relation of miR-107 to caspase-1.

**Results:**

The proliferation of chondrocytes was decreased after LPS induction and further decreased by treatment of ATP. Single LPS treatment for chondrocytes downregulated the Col II expression while upregulated the expression of IL-1β, IL-18, and MMP-13, which was further changed by ATP treatment. miR-107 expression was decreased in chondrocytes induced by LPS and further decreased in chondrocytes induced by LPS and ATP. In addition, miR-107 overexpression increased the proliferation and decreased the pyroptosis of chondrocytes induced by LPS and ATP. miR-107 overexpression upregulated the Col II expression while down-regulated the expression of IL-1β, IL-18, and MMP-13 in supernatant of chondrocytes or chondrocytes induced by LPS and ATP. miR-107 overexpression down-regulated the expression of caspase-1, c-caspase-1, GSDMD-N, and TLR4 in chondrocytes induced by LPS and ATP. Furthermore, miR-107 directly targeted caspase-1.

**Conclusions:**

miR-107 can protect against KOA by downregulating caspase-1 to decrease pyroptosis, thereby promoting collagen protein secreted by chondrocytes by down-regulating IL-1β.

## Introduction

Knee osteoarthritis (KOA) is a joint disease caused by biological factors and mechanical instability, which causes degenerative and even loss of knee function [1, 2]. Its pathological features include degeneration of articular cartilage, synovial inflammatory infiltration and secondary osteophyte formation. Patients with this disease are often accompanied by chronic pain, poor flexion and extension of knee joints, and the late course of the disease can also incur limited range of movement or even disability [3, 4]. Currently, the majority of KOA patients are the middle-aged and the elder people. The characteristics of KOA, such as the long course of disease, seriously affects the quality of life of KOA patients [5]. Therefore, improving joint function of KOA patients is the current preferred strategy for KOA treatment [6].

IL-1β is an important member of the interleukin family, synthesized and secreted mainly by activated mononuclear macrophages. IL-1β belongs to the IL-1 family and is activated by caspase-1 in the NALP3 inflammasome [7]. It has been found that trace amounts of IL-1β can be found in the synovial fluid of normal joint tissues, while IL-1β is highly expressed in the synovial fluid of osteoarthritis (OA) patients, which is positively correlated with articular cartilage injury [8]. Increased IL-1β can induce chondrocytes to produce large amounts of NO through abnormal regulation of metal matrix protease (MMPs), leading to mitochondrial dysfunction, energy depletion, and other states to induce cartilage matrix degradation [9]. In addition, IL-1 can promote the production of prostaglandin E2 (PGE2) and other inflammatory mediators in chondrocytes and synovial cells, leading to synovial inflammation and bone absorption that can accelerate the development of OA [10].

IL-1β is an important inflammatory promoter and a major product of pyroptosis. Compared to normal cartilage tissues, caspase-1 expression is significantly increased in OA cartilage tissues and caspase-1 is a key enzyme in the production of active IL-1β [11]. Both pyroptosis and OA released a large amount of inflammatory mediators, and NACHT, LRR, PYD domains-containing protein 3 (NLRP3), caspase-1, IL-18, and IL-1β played an important role in OA, indicating that pyroptosis may be involved in the occurrence and development of OA [12, 13]. miR-107 has been found to regulate OA through TNF receptor-associated factor 3 (TRAF3), phosphatase and tensin homolog deleted on chromosome ten (PTEN), and human high mobility group protein B1 (HMGB1), and HMGB1 is related to pyroptosis [14–16].

To sum up, we aimed to investigate whether miR-107 could regulate KOA through pyroptosis to affect collagen protein secreted by chondrocytes through IL-1β.

## Materials and methods

### Cell culture and cell treatment

Chondrocytes were obtained from Beijing ZEPING & Technology Co., Ltd. (Beijing, China). Chondrocytes were cultured in DMEM medium containing 20% fetal bovine serum (FBS; Gibco, USA) under 37 °C with humid atmosphere of 5% CO_2_. Second generation of chondrocytes in good condition was divided into control group, LPS group, and LPS + ATP group. Cells in control group were normally cultured. Cells in LPS group were treated with 1 μg/mL LPS for 24 h. Cells in LPS + ATP group were treated with 1 μg/mL LPS for 24 h and then treated with 5.5 mmol/L ATP for 4 h.

### CCK-8 assay

After chondrocytes were treated with LPS or LPS/ATP, chondrocytes were seeded into a 96-well plate (5000 cells/well) for 24 h culture. After the medium was changed, 10% CCK-8 solution of 100 μL was added to each well, and incubated in the incubator for 1 h. The absorbance at 450 nm was detected with a microplate microscope, and the cell proliferation rate was calculated.

### ELISA assay

Supernatant of chondrocytes was collected by centrifugal tube, which was centrifuged and transferred to sterilized tubes. Next, Col II ELISA kit, IL-1β ELISA kit, IL-18 ELISA kit, and MMP-13 ELISA kit were used to detect the secretion of Col II, IL-1β, IL-18, and MMP-13 of chondrocytes. All experimental operations were performed in accordance with the instructions.

### Cell transfection

Chondrocytes in logarithmic phase were cultured overnight in 6-well plates (5 × 10^4^ cells/well). Next day, mimic NC, miR-107 mimic-#1 and miR-107 mimic-#2 were respectively transfected into chondrocytes with Lipofectamine® 2000 reagent. After chondrocytes were treated with LPS and ATP, chondrocytes were respectively transfected with mimic NC and miR-107 mimic. Culture medium was changed 6 h after transfection, and cells were collected 24 h after transfection.

### RT-qPCR analysis

Total RNA was extracted with the trizol (Thermo Fisher Scientific, USA) method. Next, according to the instructions of the reverse transcription kit, moderate amount of RNA was reversely transcribed into cDNA, which was then mixed with the SYBR Green. Finally, cDNA was amplified with the 7500 RT-PCR system (Applied Biosystems, USA). The expression of targeted genes was calculated with the 2^−∆∆Ct^ method.

### TUNEL assay

Chondrocytes in each group with good growth after transfection were selected. Cell slide of each group was fixed with 4% polyformaldehyde for 30 min and washed with PBS for 5 min. Then, 0.1% Triton X-100 was used to penetrate the cell membranes and cells were washed with PBS again. The apoptosis of chondrocytes was studied via TUNEL Apoptosis Kit (Invitrogen), with employment of DAPI (Koritai Biotechnology, Beijing, China) for dying. Cells were then observed and captured by fluorescence microscopy (Olympus, Tokyo, Japan).

### Western blot analysis

After removing the medium, chondrocytes were washed twice with PBS and then the cells were lysed with lysis buffer. The collected proteins were determined by BCA method. Twenty micrograms of protein samples were loaded, separated with the 10% SDS-PAGE gel (Beyotime, China), and transferred to the PVDF membranes (Millipore, USA). Then, membranes were sealed with 5% skim milk for 1 h and incubated with the primary antibodies overnight at 4 °C. The primary anti-bodies used are Col II, IL-1β, IL-18, MMP13, caspase-1, c-caspase-1, GSDMD-N, TLR4, and GAPDH. On the next day, horseradish peroxidase-conjugated secondary antibodies were added to membranes for 1 h culture at 25 °C. An appropriate amount of ECL developer was added for reaction for 1 min, and the ratio of gray value between the target protein and the internal GAPDH was analyzed by Image Lab Image processing software.

### Luciferase reporter assay

A luciferase assay was performed to identify the interaction between miR-107 and caspase-1. WT-CASP1 (caspase-1) or MUT-CASP1 (caspase-1) was transfected into chondrocytes with miR-107 mimic or mimic NC with Lipofectamine 2000 Reagent (Invitrogen, Shanghai, China). Luciferase activity was detected after transfection using a dual-luciferase reporter assay system (Promega).

### Statistical analysis

Statistical analysis of measurement data was performed using SPSS 20.0 statistical software, and the mean ± standard deviation was used for measurement of data analysis. One-way ANOVA was used for comparison between groups, and LSD *t* test was used for pairwise comparison between groups. *P* < 0.05 was considered statistically significant.

## Results

### Effects of LPS/ATP induction on chondrocytes

**Fig. 1:**
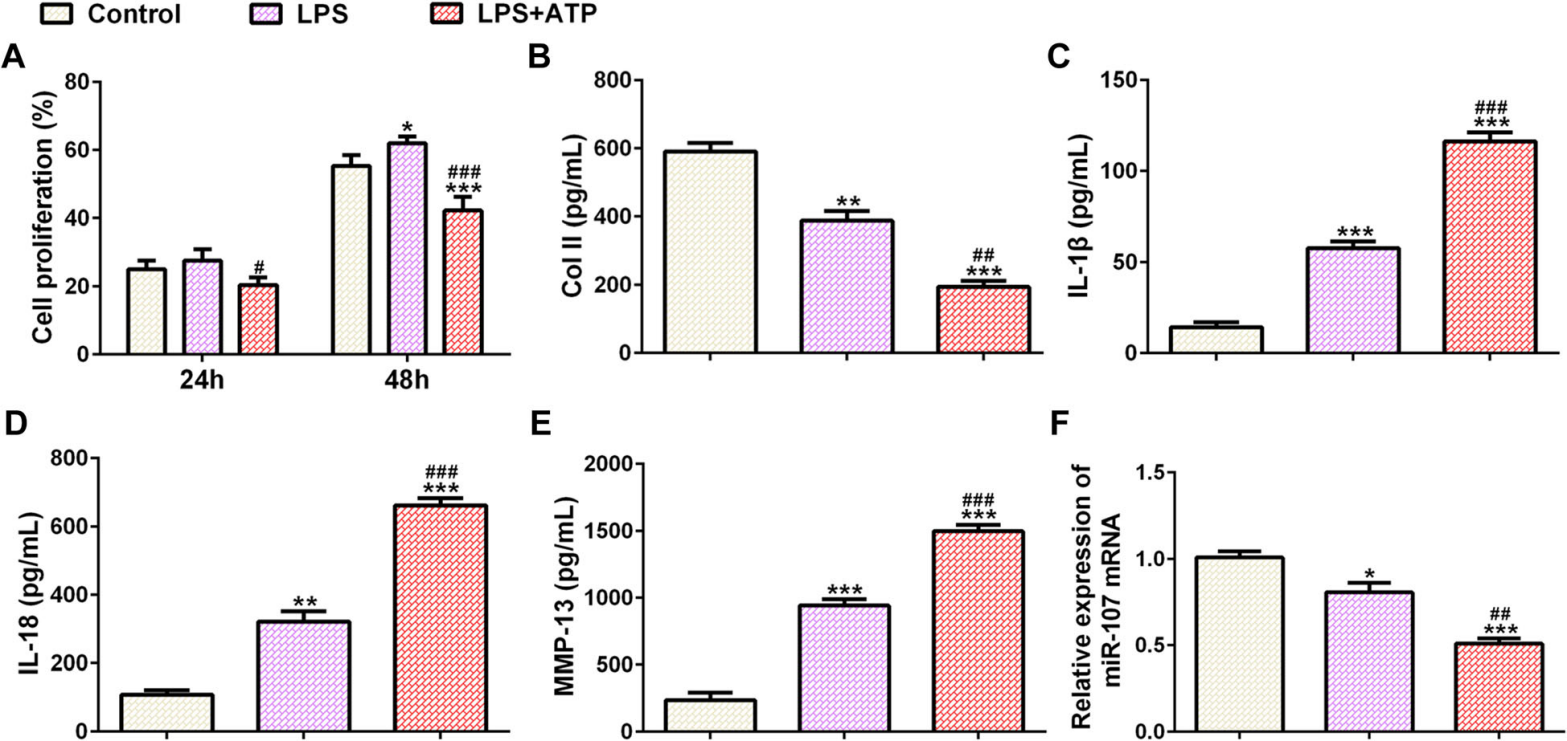
Effects of LPS/ATP induction on chondrocytes. **a** The proliferation of chondrocytes treated with LPS and ATP was detected by CCK-8 assay. *P < 0.05 and ***P < 0.001 vs. control group. ^#^*P* < 0.05 and ^###^*P* < 0.001 vs. LPS group. **b** The expression of Col II in supernatant of chondrocytes treated with LPS and ATP was detected by Col II ELISA assay. **P < 0.01 and ***P < 0.001 vs. control group. ^##^*P* < 0.01 vs. LPS group. **c** The expression of IL-1β in supernatant of chondrocytes treated with LPS and ATP was detected by IL-1β ELISA assay. ***P < 0.001 vs. control group. ^###^*P* < 0.001 vs. LPS group. **d** The expression of IL-18 in supernatant of chondrocytes treated with LPS and ATP was detected by IL-18 ELISA assay. **P < 0.01 and ***P < 0.001 vs. control group. ^###^*P* < 0.001 vs. LPS group. **e** The expression of MMP-13 in supernatant of chondrocytes treated with LPS and ATP was detected by MMP-13 ELISA assay. ***P < 0.001 vs. control group. ^###^*P* < 0.001 vs. LPS group. **f** miR-107 expression in chondrocytes treated with LPS and ATP was analyzed by RT-qPCR analysis. *P < 0.05 and ***P < 0.001 vs. control group. ^##^*P* < 0.01 vs. LPS group

After chondrocytes were treated with single LPS, the proliferation of chondrocytes was increased at 48 h. After LPS-induced chondrocytes were treated with ATP, the proliferation of chondrocytes was decreased at 24 h and 48 h (Fig. 1a). Single LPS treatment for chondrocytes downregulated the Col II expression while upregulated the expression of IL-1β, IL-18, and MMP-13. ATP treatment could further downregulate the Col II expression and upregulate the expression of IL-1β, IL-18, and MMP-13 in supernatant of LPS-induced chondrocytes (Fig. 1b). The result in Fig. 1c indicated that miR-107 expression was decreased in chondrocytes induced by LPS and further decreased in chondrocytes induced by LPS and ATP.

### miR-107 overexpression alleviates the effects of LPS/ATP induction on chondrocytes

**Fig. 2:**
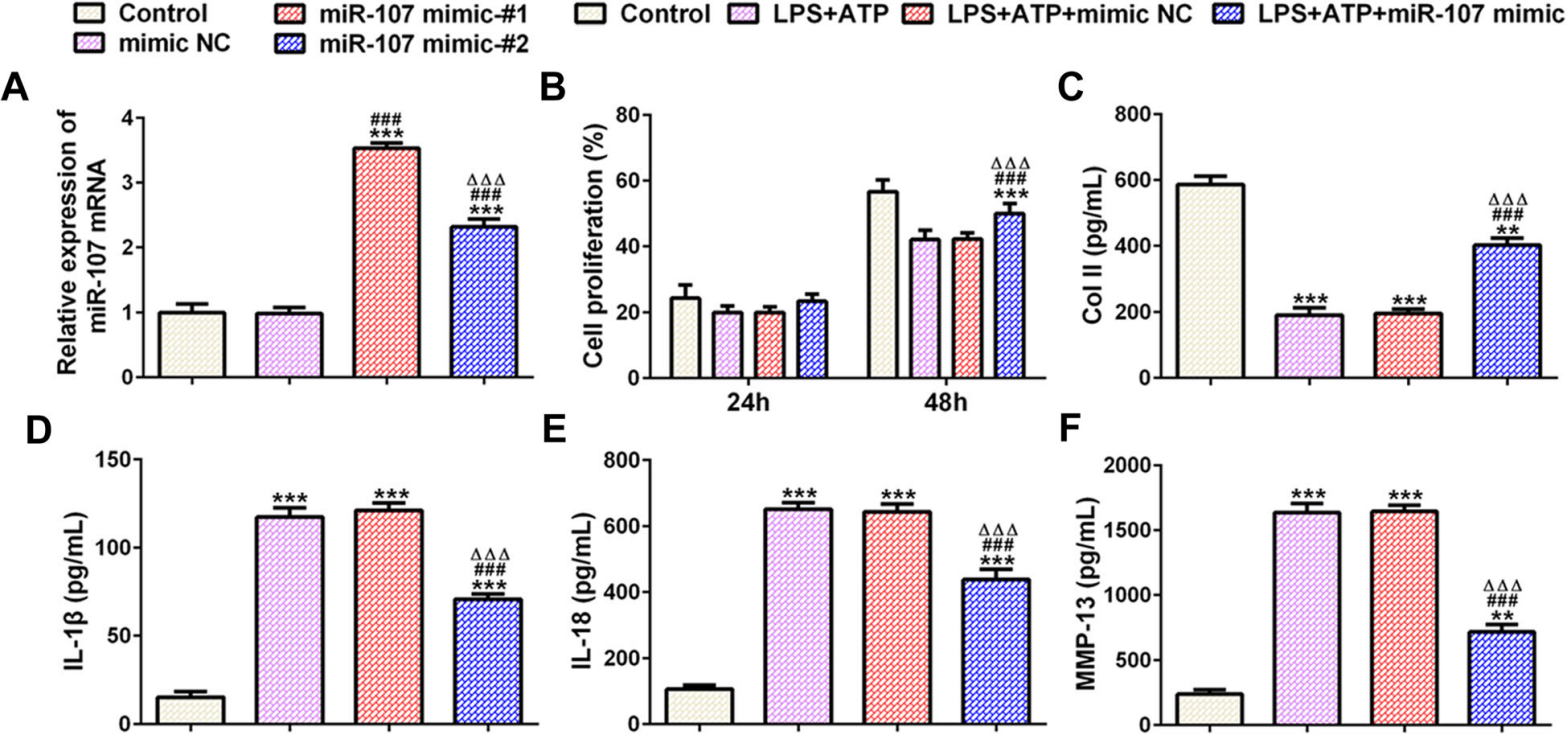
miR-107 overexpression alleviates the effects of LPS/ATP induction on chondrocytes. **a** miR-107 expression in chondrocytes treated with LPS and ATP after transfection was analyzed by RT-qPCR analysis. ***P < 0.001 vs. control group. ^###^*P* < 0.001 vs. mimic NC group. ^∆∆∆^*P* < 0.001 vs. miR-107 mimic-#1 group. **b** The proliferation of chondrocytes treated with LPS and ATP after transfection was detected by CCK-8 assay. ***P < 0.001 vs. control group. ^###^*P* < 0.001 vs. LPS + ATP group. ^∆∆∆^*P* < 0.001 vs. LPS + ATP + mimic NC group. **c** The expression of Col II in supernatant of chondrocytes treated with LPS and ATP after transfection was detected by Col II ELISA assay. **P < 0.01 and ***P < 0.001 vs. control group. ^###^*P* < 0.001 vs. LPS + ATP group. ^∆∆∆^*P* < 0.001 vs. LPS + ATP + mimic NC group. **d** The expression of IL-1β in supernatant of chondrocytes treated with LPS and ATP after transfection was detected by IL-1β ELISA assay. ***P < 0.001 vs. control group. ^###^*P* < 0.001 vs. LPS + ATP group. ^∆∆∆^*P* < 0.001 vs. LPS + ATP + mimic NC group. **e** The expression of IL-18 in supernatant of chondrocytes treated with LPS and ATP after transfection was detected by IL-18 ELISA assay. ***P < 0.001 vs. control group. ^###^*P* < 0.001 vs. LPS + ATP group. ^∆∆∆^*P* < 0.001 vs. LPS + ATP + mimic NC group. **f** The expression of MMP-13 in supernatant of chondrocytes treated with LPS and ATP after transfection was detected by MMP-13 ELISA assay. **P < 0.01 and ***P < 0.001 vs. control group. ^###^*P* < 0.001 vs. LPS + ATP group. ^∆∆∆^*P* < 0.001 vs. LPS + ATP + mimic NC group

Chondrocytes were transfected with mimic NC, miR-107 mimic-#1, and miR-107 mimic-#2, and miR-107 expression was upregulated in miR-107 mimic-#1 and miR-107 mimic-#2 groups, compared with mimic NC. miR-107 expression in miR-107 mimic-#1 was the highest (Fig. 2a). The proliferation of chondrocytes in LPS + ATP group and was decreased compared with control group. There was no obvious change in cell proliferation between LPS + ATP group and LPS + ATP + mimic NC group, and miR-107 overexpression increased the proliferation of chondrocytes induced by LPS and ATP (Fig. 2b). Treatment of LPS and ATP downregulated the Col II expression while upregulated the expression of IL-1β, IL-18, and MMP-13 in chondrocytes and chondrocytes transfected with mimic NC. miR-107 overexpression upregulated the Col II expression and down-regulated the expression of IL-1β, IL-18, and MMP-13 in chondrocytes induced by LPS and ATP (Fig. 2c).

### miR-107 overexpression decreases the pyroptosis of chondrocytes induced by LPS and ATP

**Fig. 3:**
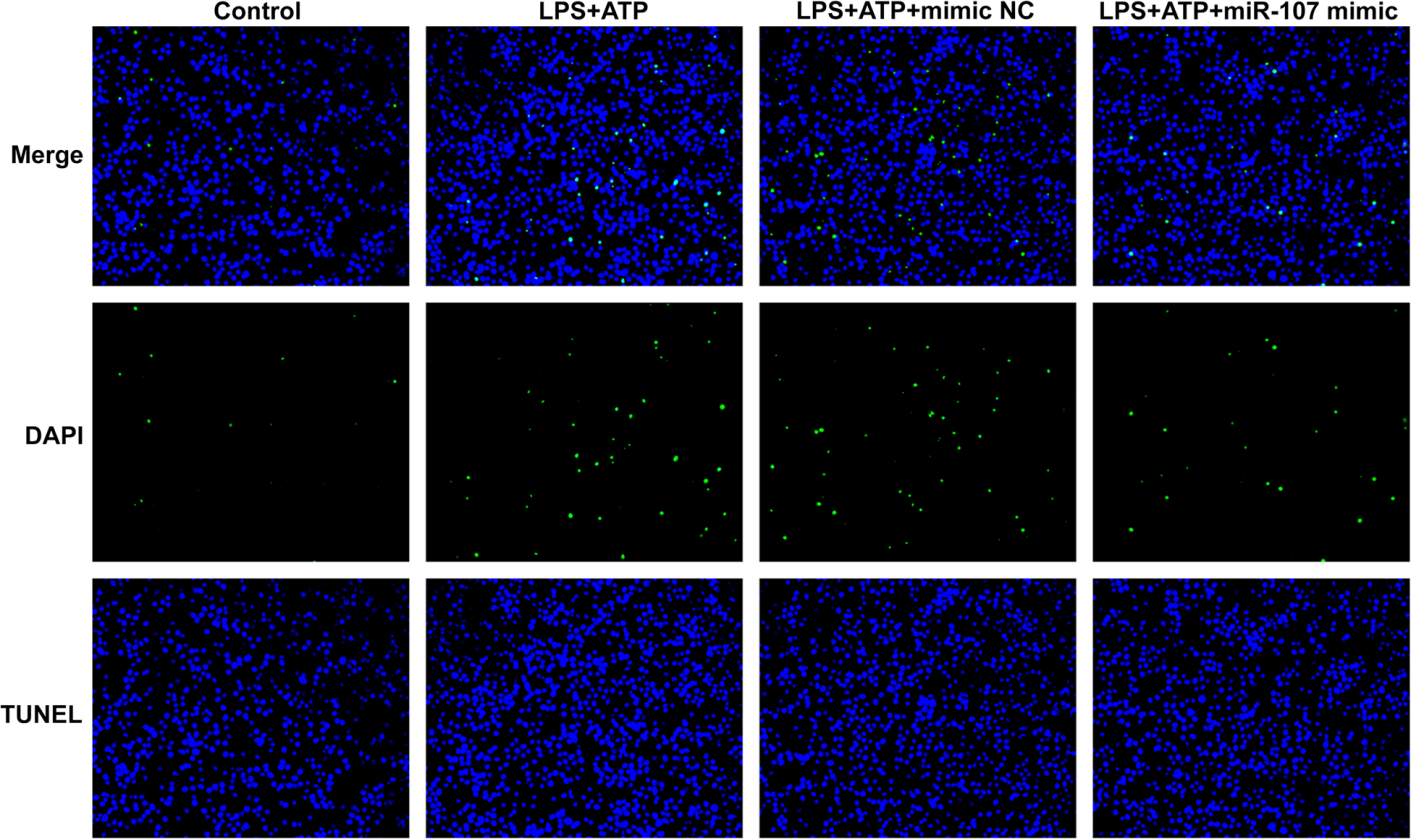
miR-107 overexpression decreases the pyroptosis of chondrocytes induced by LPS and ATP. The pyroptosis of chondrocytes induced by LPS and ATP was determined by TUNEL assay

The pyroptosis of chondrocytes in LPS + ATP group was elevated compared with control group. Mimic NC did not obviously affect the pyroptosis of chondrocytes induced by LPS and ATP. However, miR-107 overexpression decreased the pyroptosis of chondrocytes induced by LPS and ATP (Fig. 3).

### miR-107 overexpression affects the expression of cartilage matrix and pyroptosis-associated proteins in chondrocytes induced by LPS and ATP

**Fig. 4:**
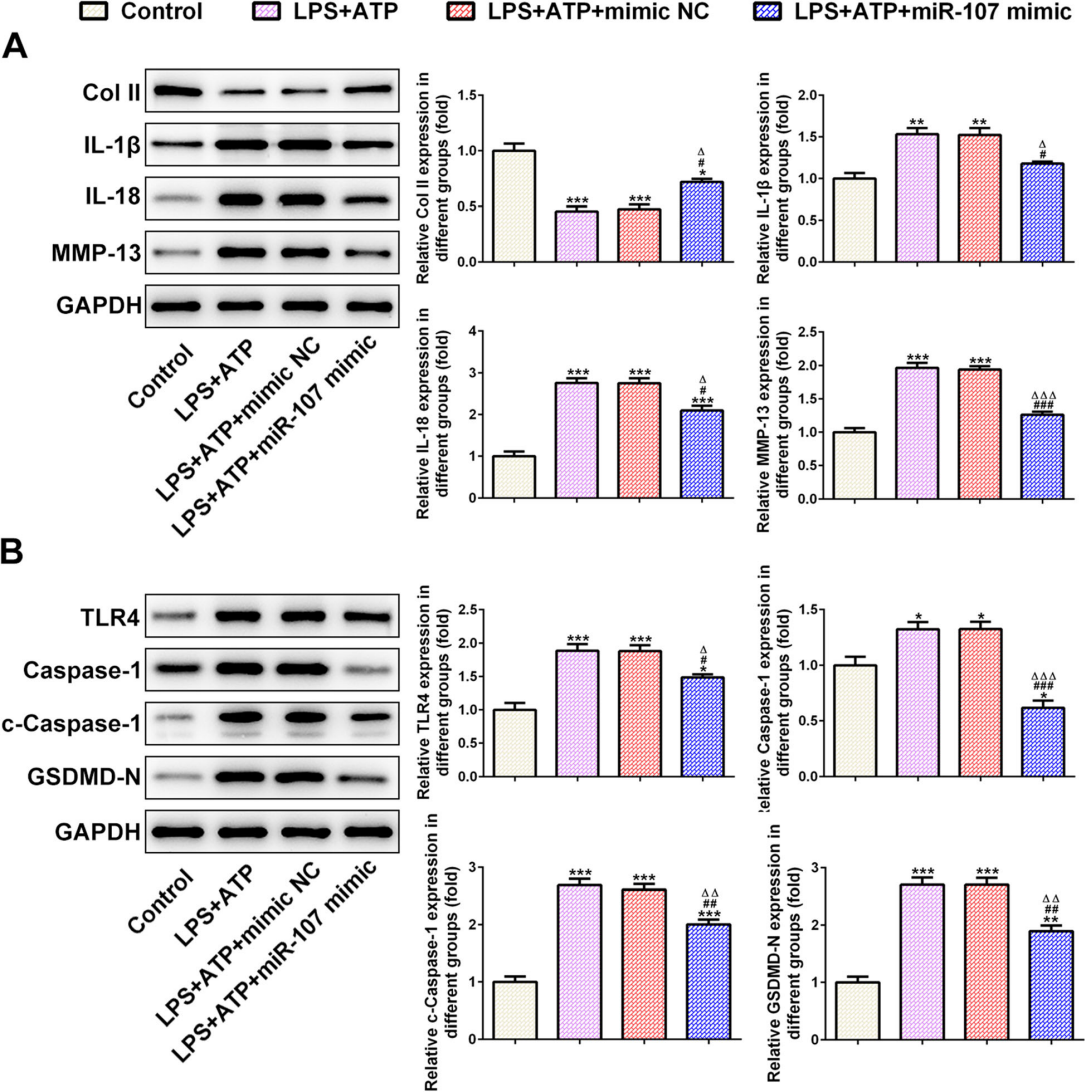
miR-107 overexpression affects the expression of cartilage matrix and pyroptosis associated proteins in chondrocytes induced by LPS and ATP. **a** The expression of Col II, IL-1β, IL-18, and MMP-13 in chondrocytes induced by LPS and ATP was detected by western blot analysis. *P < 0.05, **P < 0.01, and ***P < 0.001 vs. control group. ^#^*P* < 0.05 and ^###^*P* < 0.001 vs. LPS + ATP group. ^∆^*P* < 0.05 vs. LPS + ATP + mimic NC group. **b** The expression of caspase-1, c-caspase-1, GSDMD-N, and TLR4 in chondrocytes induced by LPS and ATP was detected by western blot analysis. *P < 0.05, **P < 0.01, and ***P < 0.001 vs. control group. ^#^*P* < 0.05, ^##^*P* < 0.01, and ^###^*P* < 0.001 vs. LPS + ATP group. ^∆^*P* < 0.05, ^∆∆^*P* < 0.01, and ^∆∆∆^*P* < 0.001 vs. LPS + ATP + mimic NC group

The Col II expression was downregulated while the expression of IL-1β, IL-18, and MMP-13 was upregulated in chondrocytes induced by LPS and ATP. Mimic NC had no obvious effect on the expression of the above proteins, and miR-107 overexpression upregulated the Col II expression while downregulated the expression of IL-1β, IL-18, and MMP-13 in chondrocytes induced by LPS and ATP (Fig. 4a). The expression of caspase-1, c-caspase-1, GSDMD-N, and TLR4 in chondrocytes induced by LPS and ATP was increased, which was not affected by mimic NC. miR-107 overexpression downregulated the expression of caspase-1, c-caspase-1, GSDMD-N, and TLR4 in chondrocytes induced by LPS and ATP (Fig. 4b).

### miR-107 directly targets caspase-1

**Fig. 5:**
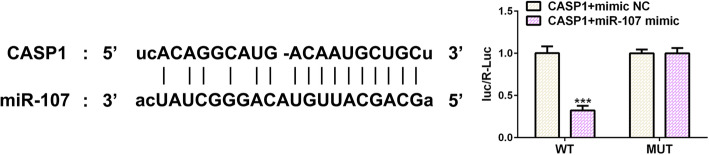
miR-107 directly targets caspase-1. The binding sites of miR-107 and caspase-1 was predicted by ENCORI and luciferase reporter assay was used to demonstrate the combination of miR-107 and caspase-1. ***P < 0.001 vs. CASP1 + mimic NC group

The relative luciferase activity was obviously decreased when cells were co-transfected with miR-107 mimic and WT-CASP1, and it was not changed in cells co-transfected with miR-107 mimic and MUT-CASP1, indicating that miR-107 directly targeted caspase-1 (Fig. 5).

## Discussion

In this study, miR-107 overexpression suppressed cartilage matrix degradation by up-regulating the Col II expression and downregulating the expression of MMP-13, which was the most active Col II lyase in chondrocytes induced by LPS and ATP. In addition, we can translate the results of basic research into real treatments for patients in the development of drugs to fill the gap between basic sciences and clinical sciences [17, 18].

The cartilage degeneration is often considered to be the main cause for the progression of KOA [19], and improvement of chondrocyte function is important for KOA recovery.

Classical pyroptosis mainly relies on caspase-1 activated by inflammasomes. Increased expression of caspase-1 is associated with NLRP3 inflammasome-mediated pyroptosis signaling [20, 21]. Rajamaki et al. [22] proved that oxidized low-density lipoprotein and cholesterin crystals activated NLRP3 inflammasome in macrophages, which further activated caspase-1 to mature pro-inflammatory factors (IL-1β and IL-18) that could induce macrophage pyroptosis. Gout arthritis is an inflammatory disease induced by sodium urate (MSU) crystals or pyrophosphate dihydrate (CPPD) crystals. MSU and CPPD were recognized by NALP3 inflammasome to activate NALP3, which then activated caspase-1 to produce mature IL-1 and IL-18 that induce inflammation [20]. At the onset of gouty arthritis, TLR4 signaling pathway was involved in regulating cell pyroptosis. TLR4 signals recognized the MSU and interacted with MyD88 to activate NF-κB, thereby regulating the expression of pro-IL-1β, which was cut into mature IL-1β to induce cell pyroptosis [23].

Activation of caspase-1 is the core of cell pyroptosis. A study showed that anti-inflammatory therapy targeting NLRP3 inflammasome could be a new approach to the treatment of OA [24]. It was reported that the expression of NLRP3, IL-18, and IL-1β in joint synovial fluid was significantly increased in mice with collagen-induced arthritis, and the expression of NLRP3 was correlated with the severity of clinical arthritis [12]. Denoble et al. [13] found that uric acid in OA patients’ synovium could activate pro-IL-18 and pro-IL-1β by activating NLRP3 inflammasome, and IL-18 and IL-1β in synovium was positively correlated with the severity of OA. Therefore, we speculated that pyroptosis might be involved in the process of OA. In this study, chondrocytes induced by LPS were used to simulate the KOA model in vitro. The expression of IL-1β, IL-18, and MMP-13 was increased in chondrocytes induced by LPS, which induced the decreased expression of Col II. The finding was consistent with the previous study [25]. In addition, ATP induction caused the increased expression of caspase-1, which could stimulate pyroptosis of chondrocytes.

Recently, miRNAs have been demonstrated to regulate the development of KOA [26–28]. miR-107 was reported to be down-regulated in OA, and miR-107 overexpression could alleviate chondrocyte damage [14–16]. In this study, miR-107 expression was decreased in chondrocytes induced by LPS and further decreased in LPS-induced chondrocytes treated with ATP. miR-107 directly targets caspase-1. miR-107 overexpression downregulated the caspase-1 expression to decrease pyroptosis and promote the proliferation of chondrocytes. Furthermore, miR-107 overexpression decreased the expression of IL-1β, IL-18, and MMP-13, thereby promoting the expression of Col II.

## Conclusion

Above all, miR-107 expression was decreased in chondrocytes induced by LPS and further decreased in LPS-induced chondrocytes treated with ATP. miR-107 overexpression promoted the proliferation and inhibited pyroptosis of chondrocytes by downregulating caspase-1 expression to alleviate inflammation, thereby promoting collagen protein secreted by chondrocytes. The newly found results will provide a novel insight into therapeutic approach to KOA treatment. However, limitations still exist in this study due to the lack of in vivo experiments. In the future, animal experiments or clinical studies will be performed to support the new findings concluded from this study.

## Data Availability

The experimental data will be available on the request.

## Abbreviations

KOA: Knee osteoarthritis
IL-1β: Interleukin-1 beta
IL-18: Interleukin-18
Col II: Collagen II
CCK-8: Cell counting kit-8
ELISA: Enzyme-linked immunosorbent assay
LPS: Lipopolysaccharide
ATP: Adenosine triphosphate
OA: Osteoarthritis
MMPs: Metal matrix protease
PGE2: Prostaglandin E2
NLRP3: NACHT, LRR, and PYD domains-containing protein 3
TRAF3: TNF receptor-associated factor 3
PTEN: Phosphatase and tensin homolog deleted on chromosome ten
HMGB1: Human high mobility group protein B1
